# Multiomics-based molecular subtyping based on the commensal microbiome predicts molecular characteristics and the therapeutic response in breast cancer

**DOI:** 10.1186/s12943-024-02017-8

**Published:** 2024-05-10

**Authors:** Wenxing Qin, Jia Li, Na Gao, Xiuyan Kong, Liting Guo, Yang Chen, Liang Huang, Xiaobing Chen, Feng Qi

**Affiliations:** 1 Department of Medical Oncology, Fudan University Shanghai Cancer Center, Shanghai, 200032 PR China; 2 Department of Oncology, Shanghai Medical College, Fudan University, Shanghai, 200032 PR China; 3 Department of Oncology, Ruijin Hospital, Shanghai Jiao Tong University School of Medicine, Shanghai, 200025 PR China; 4 Department of Thoracic Surgery, Ruijin Hospital, Shanghai Jiao Tong University School of Medicine, Shanghai, 200025 PR China; 5 Department of Laboratory Medicine, Zhongnan Hospital of Wuhan University, Wuhan University, Wuhan, 430071 PR China; 6 Zhejiang Key Laboratory of Intelligent Cancer Biomarker Discovery and Translation, First Affiliated Hospital of Wenzhou Medical University, Wenzhou, 325035 PR China; 7 Department of Oncology, The Affiliated Cancer Hospital of Zhengzhou University, Henan Cancer Hospital, No. 127, Dongming Road, Zhengzhou, 450008 PR China; 8 Department of Breast Surgery, Shanghai Medical College, Fudan University Shanghai Cancer Center, Fudan University, Shanghai, 200032 PR China

## Abstract

The gut microbiota has been demonstrated to be correlated with the clinical phenotypes of diseases, including cancers. However, there are few studies on clinical subtyping based on the gut microbiota, especially in breast cancer (BC) patients. Here, using machine learning methods, we analysed the gut microbiota of BC, colorectal cancer (CRC), and gastric cancer (GC) patients to identify their shared metabolic pathways and the importance of these pathways in cancer development. Based on the gut microbiota-related metabolic pathways, human gene expression profile and patient prognosis, we established a novel BC subtyping system and identified a subtype called “challenging BC”. Tumours with this subtype have more genetic mutations and a more complex immune environment than those of other subtypes. A score index was proposed for in-depth analysis and showed a significant negative correlation with patient prognosis. Notably, activation of the TPK1-FOXP3-mediated Hedgehog signalling pathway and TPK1-ITGAE-mediated mTOR signalling pathway was linked to poor prognosis in “challenging BC” patients with high scores, as validated in a patient-derived xenograft (PDX) model. Furthermore, our subtyping system and score index are effective predictors of the response to current neoadjuvant therapy regimens, with the score index significantly negatively correlated with both treatment efficacy and the number of immune cells. Therefore, our findings provide valuable insights into predicting molecular characteristics and treatment responses in “challenging BC” patients.

**Supplementary Information:**

The online version contains supplementary material available at 10.1186/s12943-024-02017-8.

## Introduction

The gut microbiota plays crucial roles in the occurrence, development, and treatment of diseases, including cancers [1–7]. For example, *Fusobacterium nucleatum* participates in the regulation of colorectal cancer (CRC) development [8–10], and the abundances of *Enterobacteriaceae* and *E. coli* have been demonstrated to be significantly increased in patients with inflammatory bowel disease and type 2 diabetes mellitus [11, 12]. Due to tumour heterogeneity and individual variations, tumours exhibit distinct microbial compositions, even within the same tumour type [13, 14]. This observation suggests that focusing on the microbial differences between tumours may be overly restrictive. Recent studies have shown that probiotics increase the production of short-chain fatty acids and effectively alleviate the symptoms of different diseases [3, 6, 15–17], indicating that the gut microbiota affects various diseases through common metabolites and pathways. Hence, exploring antitumour treatments based on the key metabolic pathways of the gut microbiota is a promising strategy.

Breast cancer (BC) is a malignant tumour originating from the mammary gland epithelium that often presents with inconspicuous early symptoms, making timely detection challenging [18, 19]. Statistically, 3-10% of newly diagnosed BC patients have distant metastases at the time of diagnosis [20]. Current treatments for advanced BC are typically stratified based on molecular subtype, considering the patient’s prior treatment history and therapeutic sensitivity [21–27]. Clinically, the molecular subtypes of BC are luminal A (LumA), luminal B (LumB), triple-negative breast cancer (TNBC), and HER2-positive BC, and each has distinct therapeutic approaches and efficacies [21, 23, 25–27]. The responses and outcomes of patients vary widely, even among patients with the same subtype [25, 28], suggesting that the traditional BC subtyping system may not be universally applicable. Therefore, the exploration of new BC subtyping approaches is necessary to increase the effectiveness of treatments.

To identify gut microbiota-related metabolic pathways and develop a new subtyping system for BC, we first analysed the differentially abundant genera and various metabolic pathways in BC, CRC and GC by machine learning methods. Most of the differentially abundant genera were cancer specific, and 36 metabolic pathways were shared among the three cancer types, with consistent expression trends. This finding implies that these shared metabolic pathways of the gut microbiota may play important roles in the occurrence and development of cancer. Next, based on gene expression profiles related to microbial metabolic pathways and patient prognostic data, BC patients were subtyped into four clusters. Among these clusters, the subtype represented by Cluster 2 was called “challenging BC” due to the increases in genetic mutations and the complexity of the immune microenvironment. Accordingly, a score index was developed and was found to be negatively correlated with patient survival. We found that in the low-score group, ARHGAP15 tumour cells and CD8 + CCL5 immune cells were significantly colocalized, indicating good spatial consistency, according to the spatial transcriptome sequencing (ST-seq) data. This pattern was also observed for the TPK1 tumour cells and both CD4 + FOXP3 and CD8 + CXCL13 + ITGAE immune cells in the high-score group. Pearson correlation analysis revealed a positive correlation between the number of colocalized cells in each score group. The applicability of this new subtyping method was subsequently validated by investigating the relationship between signalling pathways affected by the dominant cells in the high-score group and poor prognosis in a patient-derived xenograft (PDX) mouse model and was further supported by the significant negative correlations between the score index and both treatment efficacy and the expression of immune cells.

## Results

### Gut microbiota-related metabolic pathways in BC, CRC and GC

**Fig. 1:**
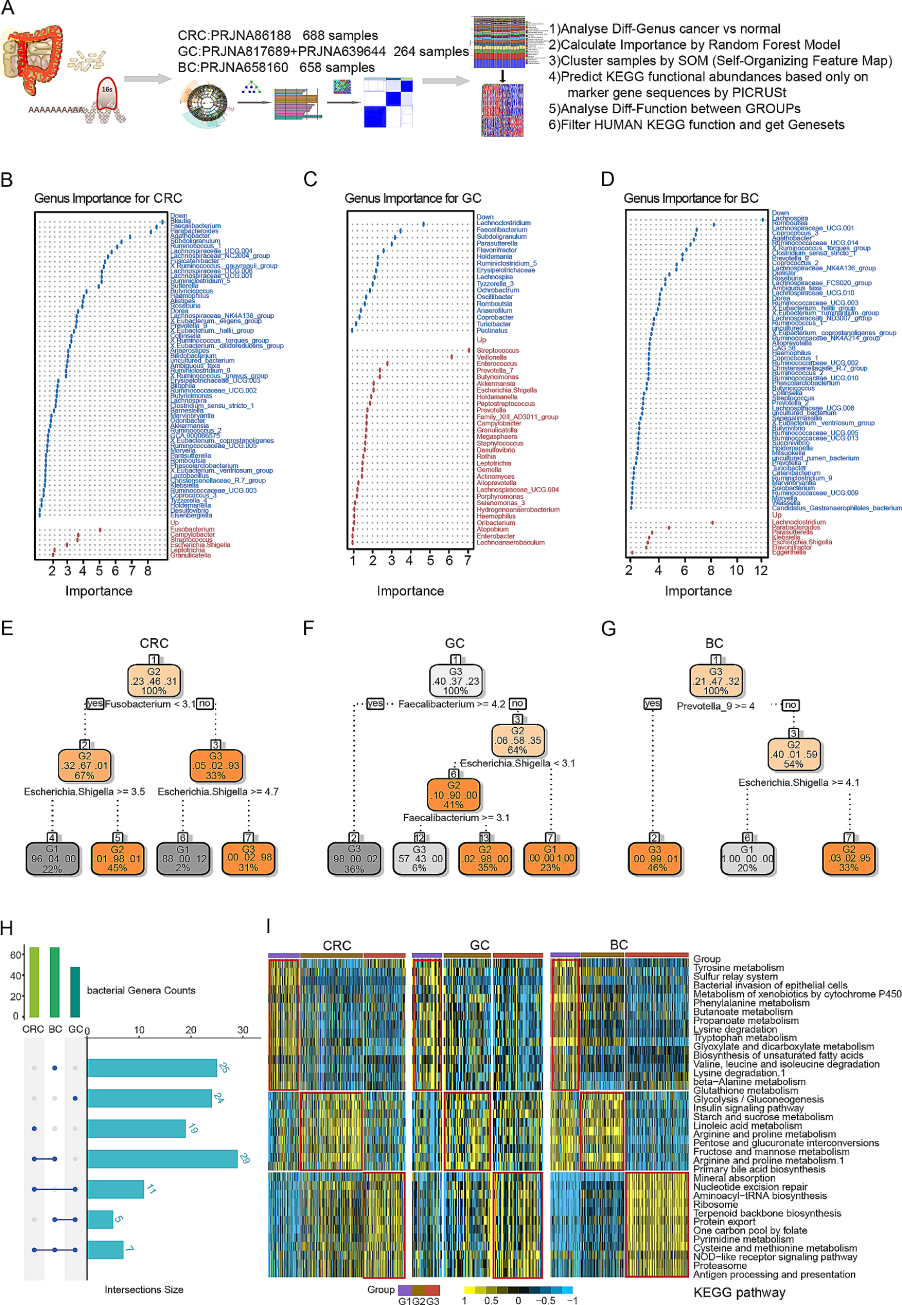
Gut microbiota characteristics and clustering analysis based on machine learning in BC, GC, and CRC patients. (**A**) Flowchart of the gut microbiota analysis. (**B**-**D**) The importance of the significantly different genera in CRC, GC and BC patients. Up: significantly upregulated genes in cancer; Down: significantly downregulated genes in cancer. (**E**-**G**) Clustering of CRC, GC and BC samples based on the self-organizing map (SOM) method. All three cancer cohorts were divided into three clusters, denoted G1, G2, and G3. *Fusobacterium* and *Escherichia Shigella* were the key genera marking the clusters of CRC patients. *Faecalibacterium* and *Escherichia Shigella* were key genera marking the clusters of GC patients. *Prevotella_9* and *Escherichia Shigella* were key genera marking the clusters of BC patients. (**H**) Venn diagram of the significantly different bacterial genera among the three cancers. The differences in the overlapping bacterial genera among the three cancers were not extensive, with nearly half of the genera unique to each cancer, a phenomenon possibly related to tumour specificity. (**I**) Heatmap of the differentially enriched metabolic pathways among the clusters in the three cancers. The microbial functions in the BC cohort were similar to those in the CRC and GC cohorts

To investigate the microbiota-related metabolic pathways shared by cancers, we analysed 16 S rRNA sequencing data of the gut microbiota obtained from four public datasets (PRJNA86188, PRJNA817689, PRJNA639644, and PRJNA658160) (Fig. 1A and Supplementary Tables 1–3). PRJNA861885 contained data for 428 CRC specimens and 260 normal samples. PRJNA817689 and PRJNA639644 contained data for 124 GC specimens and 140 normal samples, and PRJNA658160 contained data for 350 BC specimens and 308 normal samples. Using the Wilcoxon test and a random forest model, we identified significantly differentially abundant bacterial genera between the normal groups and the cancer groups (Fig. 1B-D, Methods). Based on the differentially abundant bacterial genera, all patients were clustered into three subgroups by the self-organizing map (SOM) method. Each cancer cohort was divided into the G1, G2, and G3 subgroups (Fig. 1E-F and Supplementary Table 4), which exhibited distinct gut microbiota characteristics at the phylum and genus levels (Supplementary Fig. 1A-I). For all three cancer types, the differentially abundant genera were tumour specific and enriched in different subgroups. For example, the BC cohort had 25 unique differentially abundant genera, and the GC and CRC cohorts had 24 and 19 different genera, respectively. Only seven genera were shared among the three cancers (Fig. 1H). *Escherichia Shigella* was enriched in the G1 subgroup of CRC patients, G3 subgroup of GC patients, and G2 subgroup of BC patients. These results suggest that focusing on different bacterial genera has limited the understanding of the development of different cancer types. Therefore, using Phylogenetic Investigation of Communities by Reconstruction of Unobserved States (PICRUSt) software and one-way analysis of variance (ANOVA), we identified Kyoto Encyclopedia of Genes and Genomes (KEGG) pathways that exhibited significant differential enrichment among the subgroups. We focused on 36 differentially enriched metabolic pathways shared by the three cancer types and found that their enrichment trends were consistent among the subgroups (Fig. 1I and Supplementary Table 5). For example, we observed significant alterations in cysteine and methionine metabolism in the G3 subgroups of the BC, CRC, and GC cohorts, consistent with previous reports [29, 30]. These results showed that although the microbiomes of different tumours have different microbial compositions, they have conserved effects on these 36 metabolic pathways, implying that these shared metabolic pathways may play important roles in tumour development.

### Development of the new BC subtyping method

**Fig. 2:**
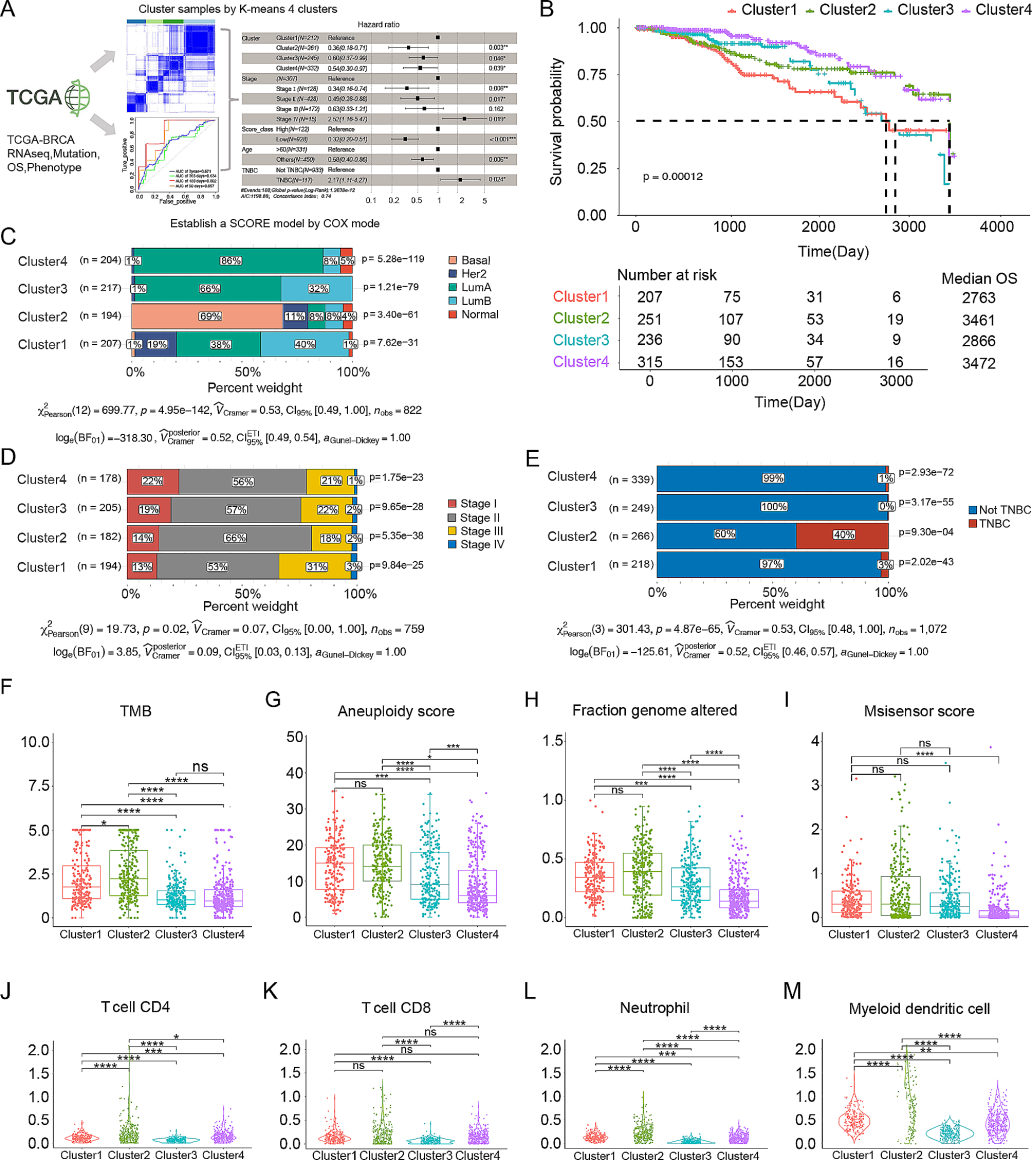
Distinct features of the BC subtypes constructed based on the commensal microbiome and metabolic pathways and genes significantly associated with survival. (**A**) Flowchart of the TCGA-BRCA dataset analysis. Based on the significance of the identified pancancer pathways, we selected genes associated with those pathways and filtered for pathways significantly correlated with survival. (**B**) Survival curves for the BC clusters. The prognostic outcomes varied significantly, with Cluster 4 displaying the best prognosis and Cluster 1 the poorest. (**C**-**E**) Proportions and chi-square test P values based on the traditional molecular subtypes, clinical stage, and TNBC status in each cluster of BC patients. (**F**-**I**) Boxplots of the TMB, aneuploidy score, fraction of genome altered, and MSIsensor score for the BC clusters at the genomic level. Cluster 2 had the highest values, and Cluster 4 had the lowest values. (**J**-**M**) Boxplots of CD4 + T-cell, CD8 + T-cell, neutrophil, and myeloid dendritic cell counts for the BC clusters at the immune level. Cluster 2 had the highest abundances among the clusters. **** *P* < 0.0001. *** *P* < 0.001. **P < 0.01. *P < 0.05. ns, *P* > 0.05

The expression of genes associated with the shared microbial metabolic pathways was then assessed. We integrated multiomics data from The Cancer Genome Atlas breast cancer (TCGA-BRCA) dataset, such as gene expression profile, clinical phenotype, RNA-seq and clinical data, to develop a new BC subtyping system. A total of 700 genes associated with gut microbiota-related metabolic pathways and patient survival were identified and used for clustering the TCGA-BRCA patients into four clusters using distance-based k-means clustering [31] (Fig. 2A and Supplementary Table 6). Each cluster exhibited distinct gene expression patterns and hallmark pathways, with Cluster 4 showing the best prognosis and Cluster 1 showing the poorest prognosis (Supplementary Fig. 2A-B and Fig. 2B). Analysis revealed significant enrichment of immune-related pathways in Cluster 4, validating the accuracy of our subtyping method (Supplementary Fig. 2C-D). Furthermore, the clusters demonstrated differences in PAM50 molecular subtyping [32] (*P* = 4.95e-142, 95% CI [0.49, 1.00]), stage distribution (*P* = 0.02, 95% CI [0.00, 1.00]), and TNBC incidence (*P* = 4.87e-65, 95% CI [0.48, 1.00]). The four clusters also exhibited different clinical characteristics (Fig. 2E-G). Cluster 2 included patients with all PAM50 molecular subtypes, such as the LumA, LumB, Her-2, basal and normal-like subtypes. Cluster 3 predominantly included patients with the LumA and LumB subtypes, and Cluster 4 consisted primarily of patients with the LumA subtype (Fig. 2E). P (Fig. 2F). Cluster 2 was significantly enriched in TNBC patients (Fig. 2G). Additionally, at the genomic level, we evaluated the tumour mutation burden (TMB), aneuploidy score, fraction of genome alterations, and MSIsensor score. At the immune level, we calculated the abundances of CD4 + T cells, CD8 + T cells, neutrophils, and myeloid dendritic cells (Fig. 2H-O). Cluster 1 and Cluster 2 had the highest TMB values, while Cluster 3 and Cluster 4, especially Cluster 4, had the lowest TMB values, consistent with the results of our previous prognostic analysis. However, notably, Cluster 2 was associated with the highest TMB value but not the worst prognosis. This discrepancy may be related to the complex immune environment of Cluster 2, as demonstrated in our findings. Overall, our multiomics-based subtyping method captures distinct molecular and immune characteristics of BC.

### The score was significantly associated with the prognosis of “challenging BC” patients

**Fig. 3:**
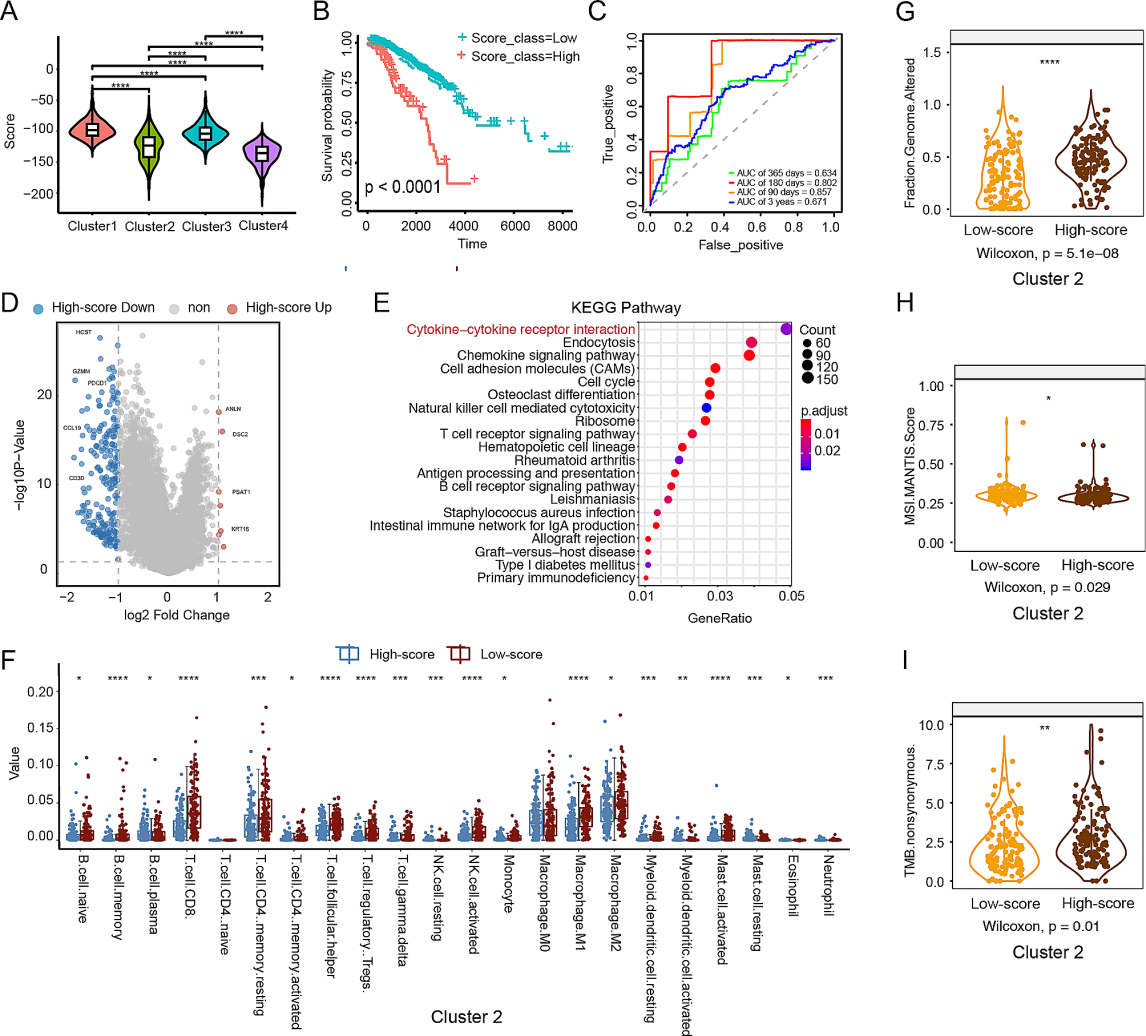
Identification and prognostic analysis of the “challenging BC” subtype and the molecular characteristics of this subtype. (**A**) Violin plots showing the distribution of scores across the four clusters. (**B**) Survival curves for the low-score and high-score groups. Patients in the high-score group exhibited poorer survival outcomes. (**C**) The predictive value of the score in the TCGA-BRCA cohort (AUCs: 0.857, 0.802, 0.634 and 0.671; 90-, 180- and 365-day OS, respectively). (**D**) Volcano plot of the DEGs between the high-score and low-score groups. Red indicates significantly upregulated genes in the high-score group, and blue indicates significantly upregulated genes in the low-score group. (**E**) Bar plot of differentially enriched pathways between the high-score and low-score groups. The pathways associated with cancer were significantly enriched in the high-score group, accompanied by significantly higher scores. (**F**) Comparison of immune cell populations in Cluster 2 between the high-score and low-score groups. (**G**-**I**) Boxplots showing the fraction of genome alterations, TMB, MSI MANTIS score and nonsynonymous TMB between the high-score and low-score groups for the BC clusters at the genomic level. The high-score group in Cluster 2 had significantly greater values of these parameters, indicating a greater mutation burden in patients in the high-score group. *** *P* < 0.001. **P < 0.01. *P < 0.05

A new subtype of BC, termed “challenging BC”, was identified using the novel BC subtyping method developed in this study. Cluster 2 exhibited more genetic mutations and a more complex immune microenvironment than did the other clusters, leading to its designation as the “challenging BC” subtype. Cluster 2 contained patients with all of the traditional subtypes, including the LumA, LumB, Her-2-positive, basal, normal-like, and TNBC subtypes (Fig. 2C and E). Notably, TNBC patients were significantly overrepresented in Cluster 2. The inherent complexity of treatment for Cluster 2 patients poses substantial challenges and underscores the clinical significance of this subtype. To further analyse the “challenging BC” subtype, a score index was proposed based on gene expression and its independent prognostic coefficient (Methods). Each patient was assigned a score, and Cluster 2 exhibited the highest degree of score dispersion (Fig. 3A and Supplementary Table 7). Patients were then divided into the high-score and low-score groups based on the median score, and analysis revealed significantly poorer survival outcomes in the high-score group (Fig. 3B). Moreover, the score index emerged as an independent prognostic factor, with area under the receiver operating characteristic (ROC) curve (AUC) values of 0.634 and 0.671 for 365-day and 3-year overall survival (OS), respectively (Fig. 3C and Supplementary Fig. 3A). Subsequent analysis demonstrated significant enrichment of cancer-related and immune-related pathways in the high-score group within Cluster 2, confirming the association of Cluster 2 with poorer survival (Fig. 3D-E). Furthermore, notable differences between the high- and low-score groups in Cluster 2 were observed at both the immune and genomic levels, surpassing the differences observed in the other clusters (Fig. 3F-I and Supplementary Fig. 3B-G). These findings underscore the utility of the score index as an independent prognostic factor for the “challenging BC” subtype.

### Single-cell expression atlas and cell type identification in “challenging BC”

**Fig. 4:**
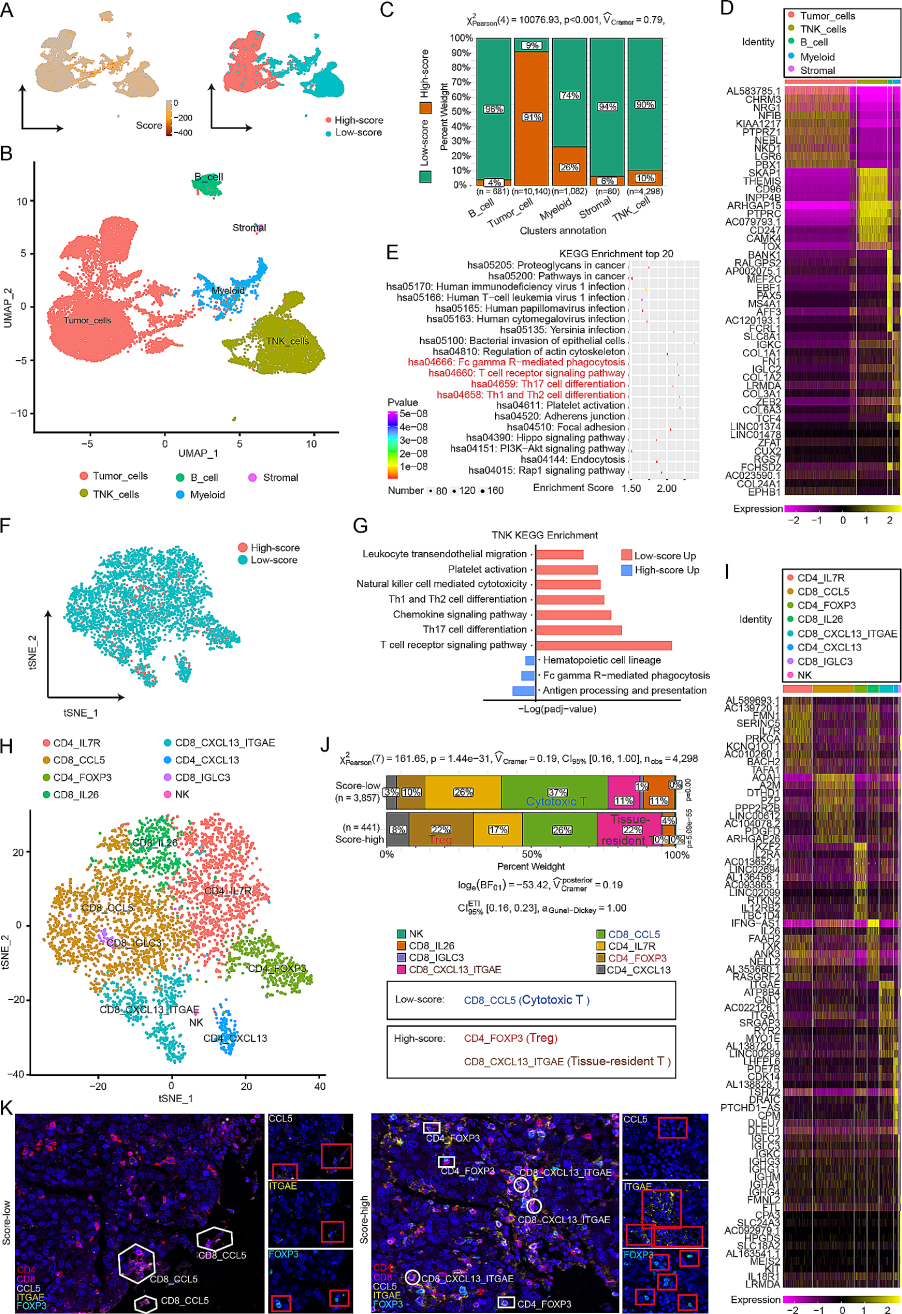
Analysis of relevant factors in “challenging BC” at the single-cell level. (**A**) Uniform manifold approximation and projection (UMAP) plot showing the scores in all the clusters of single-cell sequencing data. (**B**) UMAP plot showing the 5 cell types identified by integrated analysis of all the clusters. (**C**) Heatmap showing the expression of marker genes in the indicated cell types. The bar across the top labels the clusters corresponding to specific cell types. (**D**) Bar plot sho wing the percentages of the annotated cell types derived from samples with high scores and samples with low scores. I Bubble charts showing the KEGG enrichment of the DEGs between the high-score group and low-score group in all clusters. (**F**) T-distributed stochastic neighbour embedding (t-SNE) plot showing the scores for the TNK cell types. (**G**) Bar graphs showing the KEGG enrichment of the TNK cell types in the high-score group and low-score group. (H) t-SNE plot showing 8 cell types identified by integrated analysis of the TNK cell types. (**I**) Heatmap showing the expression of marker genes in the TNK cell types. The bars on the left label the clusters corresponding to specific cell types. (**J**) Bar plot indicating the percentages of annotated TNK cell types derived from samples in the high-score and low-score groups. (K) Representative multispectral images of 5 markers in tumour tissues. DAPI: cyan; CD4: red; CD8: purple; CCL5: pink; ITGAE: yellow; and FOXP3: blue

To explore immune cells linked to the prognosis of “challenging BC”, we analysed single-cell sequencing data from two patients with “challenging BC”, both of whom were pathologically diagnosed with TNBC (Fig. 4A and Supplementary Table 8). Initially, we constructed a classification model by leveraging the random forest algorithm, integrating the expression profiles of 700 genes with the classification data shown in Fig. 2. We then meticulously screened the expression of these 700 genes in each cell through single-cell sequencing and determined the average expression level of each gene. With this model, we were able to predict the subtype of the samples and assign a score to each cell based on the patient’s prognosis. The patients were then stratified into the high-score and low-score groups, and the model proficiently identified them as having the “challenging BC” subtype (Fig. 4A, Methods). We categorized the 16,282 cells that passed quality control into five major cell types (Fig. 4B and Supplementary Fig. 4A-C): tumour cells, three types of immune cells (natural killer T [TNK] lymphocytes, B cells, and myeloid cells), and stromal cells. Notably, a significant proportion of tumour cells exhibited higher scores than did cells of the other types (*P* = 0.00, 95% CI [0.69, 1.00], Fig. 4C). Each cell type exhibited the expression of its well-known marker genes with high specificity (Fig. 4D). Pathway enrichment analysis based on the high- and low-score groups revealed significant differences in immune-related pathways, particularly T-cell-related pathways (Fig. 4E). Further comparison of the pathways of TNK cells revealed enrichment of immune-related pathways in the low-score group (Fig. 4F-G). TNK cells were clustered into eight distinct subtypes. CD8 + CCL5 cells, which are cytotoxic T cells, were more abundant in the low-score group and were potentially associated with a better prognosis [33, 34] (*P* = 1.44e-31, 95% CI [0.16, 1.00], Fig. 4H-J). Conversely, CD4 + FOXP3 cells, representing Treg cells, and CD8 + CXCL13 + ITGAE cells, representing tissue-resident T cells, were more prevalent in the high-score group, possibly contributing to the poorer prognosis observed in this group [35] (Fig. 4J). The results of the multicolour immunofluorescence experiments confirmed these findings (Fig. 4K), suggesting that specific immune cell populations are associated with the prognosis of “challenging BC”.

### The spatial transcriptome data suggest pathways associated with poor prognosis

**Fig. 5:**
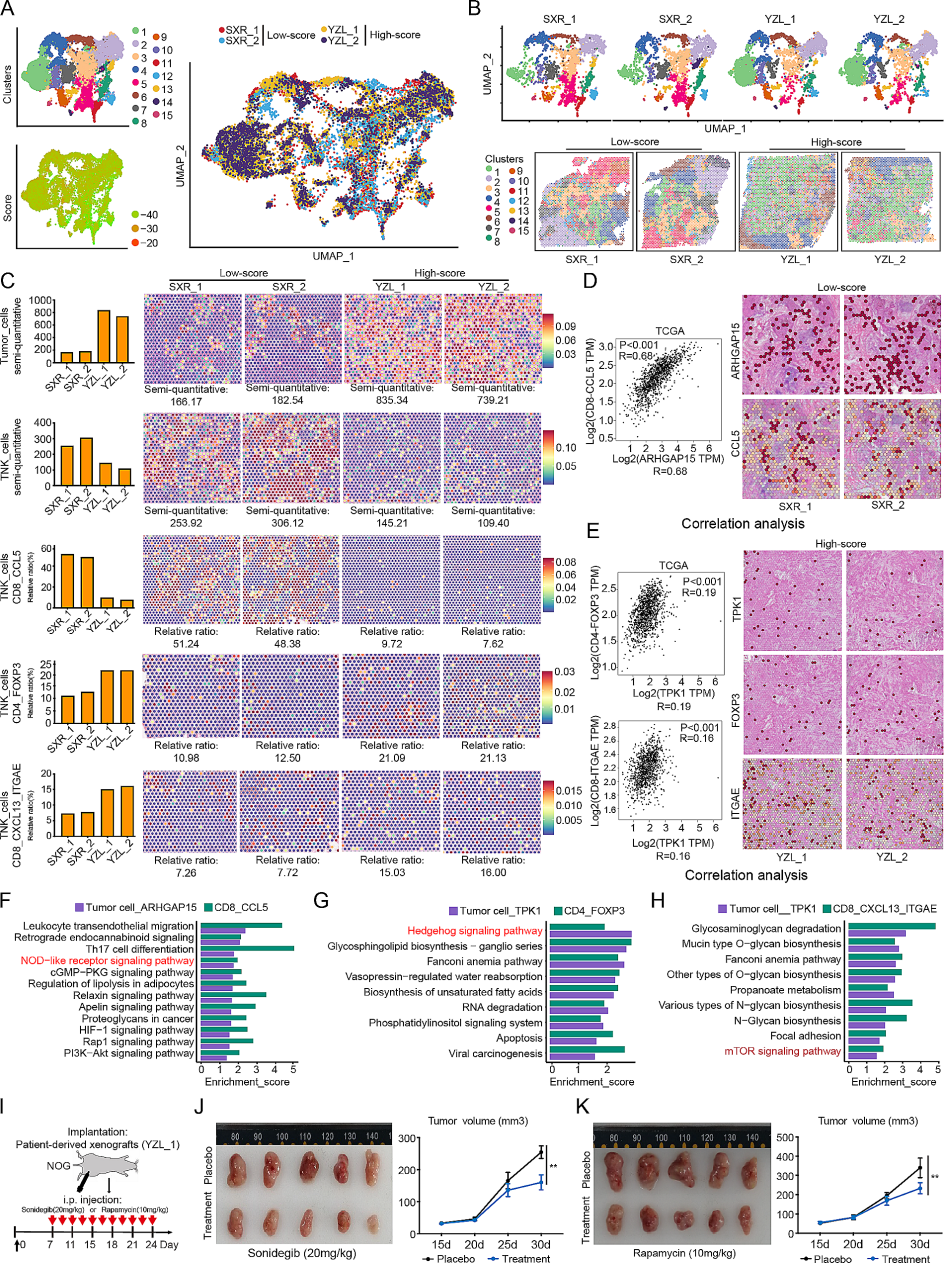
Single-cell spatial transcriptome analysis of “challenging BC” patients. (**A**) UMAP plot demonstrating the cell distribution and score variance in 4 primary tumour tissues, colour-coded by the annotated cell type and score group. (**B**) UMAP plots and spatial feature plots demonstrating the cell distribution in every tumour tissue, colour-coded by the annotated cell type. (**C**) Bar charts and spatial feature plots showing the differences in the percentages of tumour cells, TNK cells, CD8 + CCL5 cells, CD4 + FOXP3 cells, and CD8 + CXCL13 + ITGAE cells between the selected tissue sections. (**D**) Scatter plots and spatial feature plots showing the relationships between ARHGAP15 tumour cells and CD8 + CCL5 immune cells. The scatter plots were generated with data from the TCGA cohort. (**E**) Scatter plots and spatial feature plots showing the relationships among TPK1, FOXP3 and ITGAE. The scatter plots were generated with data from the TCGA cohort. (**F**) Bar graphs showing the pathways associated with the differences identified by KEGG analysis between ARHGAP15 tumour cells and CD8 + CCL5 immune cells. (**G**) Bar graphs showing the pathways associated with the differences identified by KEGG analysis between TPK1 + tumour cells and CD4 + FOXP3 immune cells. (**H**) Bar graphs showing the pathways associated with the differences identified by KEGG analysis between TPK1 + tumour cells and CD8 + CXCL13 + ITGAE immune cells. (**I**) Schematic diagram showing the experimental procedure for the implantation of patient-derived xenografts (YZL-1) into NOG mice injected with placebo, sonidegib (20 mg/kg), or rapamycin (10 mg/kg) (*n* = 5 mice per group). Student’s test; ***P < 0.001. The data are presented as the means ± SDs. (**J**) Tumour images and tumour volume curve showing the changes in tumour volume after treatment with sonidegib (20 mg/kg). Student’s test; ***P < 0.001. The data are presented as the means ± SDs. (**K**) Tumour images and tumour volume curve showing the changes in tumour volume after treatment with rapamycin (10 mg/kg). Student’s test; ***P < 0.001. The data are presented as the means ± SDs.

Spatial information plays a crucial role in the understanding of transcriptional heterogeneity and the cellular spatial distribution. In this study, we utilized ST-seq to acquire in situ gene expression profiles from four patients with “challenging BC”. The quality control results indicated significant differences among the 15 identified clusters (Supplementary 5A-C and Fig. 5A). Using a classification model and calculation methods, all four patients were identified as “challenging BC” patients who were pathologically diagnosed with TNBC. Two patients (SXR_1 and SXR_2) belonged to the low-score group, and the other two patients (YZL_1 and YZL_2) belonged to the high-score group (Fig. 5A). Although all 15 cell types were detected in each patient, their proportions and marker gene expression levels varied greatly (Fig. 5B and Supplementary Fig. 5D-F). Malignant tumour cells were more abundant in the high-score group, whereas the low-score group exhibited greater abundances of immune cells, especially cytotoxic immune cells, such as cytotoxic (CD8+) T cells, consistent with the results of prognostic analysis and single-cell sequencing (Fig. 5C, Supplementary Fig. 4D-E and Supplementary Fig. 5G-H). Specifically, the proportion of CD8 + CCL5 cells was greater in the low-score group, and CD4 + FOXP3 and CD8 + CXCL13 + ITGAE cells were more prevalent in the high-score group. These findings are consistent with previous findings (Figs. 4J-K and 5C). According to the single-cell sequencing results, most of the tumour cells in both the low- and high-score groups were ARHGAP15 and TPK1 cells, respectively (Supplementary Fig. 4F). Moreover, significant colocalization of tumour cells with specific types of immune cells was observed (ARHGAP15 cells and CD8 + CCL5 cells in the low-score group; TPK1 and both CD4 + FOXP3 and CD8 + CXCL13 + ITGAE cells in the high-score group), demonstrating spatial consistency with the ST-seq data (Fig. 5D). Pearson correlation analysis revealed positive correlations between gene expression in these cells (CD8 + CCL5 and ARHGAP15, *p* value = 0 [Fig. 5D]; TPK1 and both CD4 + FOXP3 and CD8 + ITGAE, *p* values = 1.6e-10 and 6.8e-08 [Fig. 5E]), further suggesting the influence of tumour cell populations on T-cell enrichment and the immune microenvironment. Analysis of the shared immune signalling pathways associated with prognosis identified potential pathways related to good (NOD-like receptor signalling) and poor (Hedgehog and mTOR signalling) prognoses in the low- and high-score groups, respectively (Fig. 5F-H). To validate these findings, we established a PDX model using tumour tissues from a patient in the high-score group and treated the mice with inhibitors targeting the identified signalling pathways (Fig. 5I). The inhibitors used were sonidegib and rapamycin, which are a clinically approved SMO inhibitor that inhibits Hedgehog signalling pathway activity [36–38] and an immunosuppressive mTOR inhibitor [39, 40], respectively. Significant inhibition of tumour growth was observed (Fig. 5J-K), and the identified pathways were suppressed, confirming the importance of the immune signalling pathways associated with poor prognosis identified by the score index.

### Use of the subtyping method and score index for neoadjuvant therapy

**Fig. 6:**
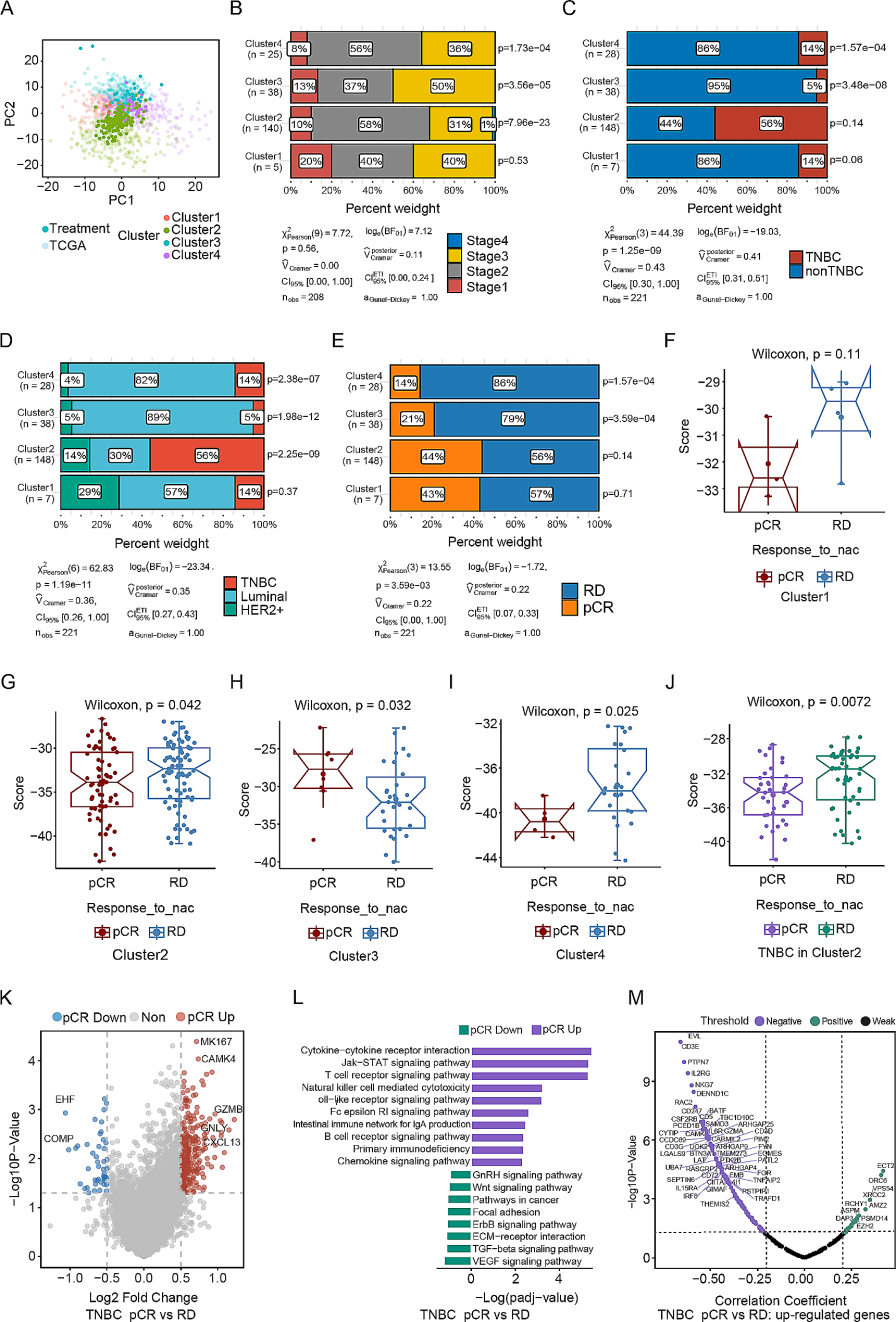
Patients with different molecular subtypes exhibit varied responses to and efficacies of neoadjuvant therapy. (**A**) Principal coordinate analysis was performed based on the Bray‒Curtis distance matrix generated from the clusters in the neoadjuvant therapy cohort. We selected 221 samples from patients who underwent neoadjuvant therapy (anthracycline and/or taxane-based therapy) to assess the impact of molecular subtype on treatment response and efficacy. (**B**-**E**) Proportions and chi-square P values for stage (*P* = 0.56, 95% CIs [0.00, 1.00]), TNBC status (*P* = 1.25e-09, 95% CIs [0.30, 1.00]), traditional molecular subtype (*P* = 1.19e-11, 95% CIs [0.26, 1.00]) and therapeutic response (*P* = 3.59e-03, 95% CIs [0.00, 1.00]) in each cluster. Upon molecular subtyping and evaluation of molecular features, we found consistent results in the new dataset, with Cluster 2 showing enrichment in TNBC samples compared with samples of other subtypes. (**F**-**J**) Boxplots showing the difference in the score between the pCR and RD groups in each cluster and in TNBC samples in Cluster 2. The four clusters exhibited different responses to treatment, with significantly higher scores in the RD group in Clusters 1, 2, and 4 (but not in Cluster 3). Similar patterns were observed in the TNBC samples. (**K**) Volcano plot of the differentially expressed genes between the pCR and RD groups in TNBC samples. Genes significantly upregulated in the pCR group are shown in red, and those significantly upregulated in the RD group are shown in blue. (**L**) Bar plot of differentially enriched pathways between the pCR and RD groups in TNBC samples. Immune activation-related pathways were significantly enriched in the pCR group. (**M**) Correlation analysis between the highly expressed genes in the pCR group and the score revealed a significant negative correlation, indicating that as the score decreased, immune-activating gene expression increased. *** *P* < 0.001. **P < 0.01. *P < 0.05

Our subtyping method and score index were also validated in a neoadjuvant therapy dataset containing data for 221 patients who received anthracycline and/or taxane-based therapy [41]. We used the classification model to determine molecular subtypes using gene expression profiles, integrating these data with survival data to derive the corresponding scores (Methods). Our objective was to assess the impact of molecular subtype on the treatment response and treatment efficacy (Supplementary Table 9). Although we observed no significant difference in the score distribution between the residual disease (RD) and pathologic complete response (pCR) groups (Supplementary Fig. 6A), the molecular subtyping method and the evaluation of molecular features yielded consistent results in this dataset (Fig. 6A-E). The four clusters exhibited varied responses to treatment, with the RD samples in Clusters 1, 2, and 4 (but not those in Cluster 3) having significantly higher scores (Fig. 6F-I). This pattern underscores the utility of our novel molecular subtyping method for BC, as it correlates the score index with treatment efficacy, indicating that higher scores are associated with lower treatment efficacy. Similar results were observed for the TNBC subset (Fig. 6J and Supplementary Fig. 6B-C). Furthermore, we analysed differential gene expression and pathway enrichment between the pCR and RD groups of TNBC patients in Cluster 2 (Fig. 6K-L). Immune activation-related pathways, such as Cytokine − cytokine receptor interaction, T-cell receptor signalling pathway, and Natural killer cell-mediated cytotoxicity, were significantly enriched in the pCR group (Fig. 6L). Correlation analysis between the highly expressed genes in the pCR group and the scores revealed a significant negative correlation, suggesting that the expression of immune-activating genes, such as NKG7, CD3E, CD247, GZMA, and IL6R, increased with decreasing score (Fig. 6M). These results indicate that the score index calculated using our subtyping method can serve as an indicator of the immune microenvironment and predict the treatment efficacy and response in BC patients receiving neoadjuvant therapy.

## Discussion

In this study, for the first time, we successfully developed a novel subtyping system for BC that integrates information on the gut microbiota, human gene expression, and patient prognosis. Through this system, we introduced a new subtype termed “challenging BC”, characterized by the presence of more genetic mutations and a more complex immune environment than other subtypes. Furthermore, we introduced a score index associated with patient prognosis, enabling the identification of “challenging BC” cases and the prediction of therapeutic responses in BC patients. The association between the classification system and the score index is hierarchical and progressive. The classification system was employed to identify cases of “challenging BC”, and the score index was subsequently utilized to conduct an in-depth analysis of these “challenging BC” cases. In this study, we leveraged multiomics data based on the gut microbiome acquired through machine learning methods, providing a scientific foundation for predicting molecular characteristics and treatment responses in patients with “challenging BC”.

Recent studies have revealed a strong association between the gut microbiota and BC [19, 42–44]. Microbial dysbiosis has been implicated in influencing the incidence of various BC subtypes. Notably, cancer patients exhibit more significant alterations in the gut microbiota composition than patients with benign tumours [45]. The gut microbiota can impact the metabolism of oestrogen and progesterone, thereby differentially affecting the incidence of steroid hormone receptor-positive and steroid hormone receptor-negative BC [46]. Hence, investigations of the mechanisms that improve the gut microbiota composition hold promise for improving the survival outcomes of BC patients and optimizing anticancer therapies. In our study, we employed machine learning methods to analyse gut microbiome data from BC, GC, and CRC patients. Although differences in the microbial genera have been implicated in different cancers, the metabolic pathways in which they participated were notably consistent. Shared metabolic pathways were observed across the BC, GC, and CRC subgroups, highlighting the importance of gut microbiota-related metabolic pathways in cancer development.

Several research groups have developed TNBC-specific subtyping systems, each including a different number of subtypes. For instance, Lehmann et al. described six subtypes [47], Burstein et al. identified four subtypes [48], Jézéquel et al. identified three subtypes [49], and Jiang et al. proposed four subtypes [50]. However, the inconsistency in results across these studies, likely stemming from differences in algorithms and patient populations, may limit the practical clinical utility of these systems. Moreover, recent findings indicate that non-TNBC tumours exhibit a limited response to treatment. For instance, sensitivity to treatment varies based on the lymphocyte concentration in Her-2-positive tumours [51–54], highlighting the heterogeneity within this subtype. These limitations underscore the need for exploring new BC subtyping approaches to improve treatment outcomes. Traditionally, BC subtyping has relied on gene expression characteristics, a strategy that also has limitations. To address this issue, we challenged the traditional BC subtyping approach and established a new system based on the gut microbiota, human gene expression profiles, and clinical features. Our novel BC subtyping method, which is based on integrated multiomics data, demonstrated applicability and accuracy across various datasets, including single-cell sequencing, single-cell spatial transcriptome, and neoadjuvant therapy datasets.

In summary, we employed machine learning methods to develop a novel BC subtyping method that integrates the gut microbiota, human genetics, and patient prognosis. This innovative approach can be used to predict not only molecular subtypes but also the prognosis of BC patients. Importantly, its applicability extends beyond BC to other types of cancers, demonstrating its universality. This method provides valuable insights for cancer treatment, particularly in addressing challenging cancer cases. However, notably, our system does not entirely replace gene expression data. Noninvasive gut microbiome data alone cannot be relied upon for direct subtyping, prognostic prediction, or treatment response assessment. This limitation of our study is acknowledged, and we aim to address it in future research efforts.

## Conclusion

In this study, for the first time, we established a groundbreaking subtyping system for BC that integrates the gut microbiota, human gene expression patterns, and patient prognosis, enabling the prediction of molecular characteristics and treatment responses. A novel subtype characterized by an increase in genetic mutations and a highly complex immune environment was identified and termed “challenging BC”. Additionally, a score index related to patient prognosis was developed, facilitating the identification of “challenging BC” cases and the prediction of therapeutic responses in BC patients. Overall, we leveraged multiomics data analyses based on the gut microbiome using machine learning methods to provide a robust scientific foundation for predicting molecular characteristics and treatment responses in patients with “challenging BC”.

### Methods

#### Sample collection

We analysed 16 S rRNA sequencing data of the gut microbiome collected from four public datasets (PRJNA86188, PRJNA817689, PRJNA639644, and PRJNA658160). PRJNA861885 included 260 normal samples and 428 CRC samples. PRJNA817689 and PRJNA639644 included 140 normal samples and 124 GC samples, and PRJNA658160 included 308 normal samples and 350 BC samples.

The mRNA expression data, clinical information, and survival data for BC patients were obtained from two publicly available data platforms: the TCGA database (https://gdc-portal.nci.nih.gov/) and the UCSC Xena Browser website (https://xenabrowser.net/datapages/). Tumour microenvironment (TME) data were obtained from Tumor IMmune Estimation Resource (TIMER) 2.0 (https://cistrome.shinyapps.io/timer/).

The neoadjuvant therapy data were obtained from the National Center for Biotechnology Information (NCBI) Gene Expression Omnibus (GEO) public database (GSE163882).

A total of 6 patients with BC were enrolled from the Department of Breast Surgery, Fudan University Shanghai Cancer Center, Shanghai Medical College, Fudan University (Shanghai, P. R. China) in 2022. Fresh breast tumour tissues were collected for single-cell transcriptome analysis (2 samples) and single-cell spatial transcriptome analysis (4 samples). All diagnoses of BC were based on histopathology and were made in accordance with the World Health Organization criteria. Ethical approval for the study was obtained from the Fudan University Shanghai Cancer Center Ethics Committee. Our single-cell transcriptome data and single-cell spatial transcriptome data have been deposited in NCBI BioProjects GSE252175 and GSE252176.

#### Microbiome analysis

The raw sequencing data were in FASTQ format. Paired-end reads were then preprocessed using Trimmomatic software [55] to detect and trim ambiguous (N) bases. Low-quality sequences with an average quality score of less than 20 were also removed using the sliding window trimming approach. After trimming, paired-end reads were assembled using FLASH software [56]. The parameters used for assembly were as follows: 10 bp of minimal overlap, 200 bp of maximum overlap and a 20% maximum mismatch rate. Further denoising was performed on the sequences as follows: reads with ambiguous sequences, homologous sequences, or fewer than 200 bp were removed; reads in which 75% of the bases had a quality score of more than 20 (Q20) were retained; and chimaeric reads were then detected and removed. These steps were performed using QIIME software [57] (version 1.8.0).

The clean reads were subjected to removal of primer sequences and clustering to generate operational taxonomic units (OTUs) using Vsearch software [58] with a cutoff of 97% similarity. The representative read of each OTU was selected using the QIIME package. All representative reads were annotated and BLASTed against the Silva database version 123 (or Greengenes) (16 S/18S rDNA) using the Ribosomal Database Project (RDP) classifier [59] (confidence threshold, 70%). All representative reads were annotated and searched against the Unite database (ITS rDNA) using BLAST [60].

Clusters were then identified. Based on the identified differentially abundant genera between normal and cancer samples, we further employed a random forest model to assess the importance of the genera, selecting those with an importance greater than the average value to obtain the final genus information [61]. Using the SOM neural network [62] library (Kohonen), we determined the optimal number of clusters and performed clustering of the cancer samples. For each identified cluster type, we used a decision tree classifier to establish classification rules.

To identify human-related genes via KEGG, we used PICRUSt [63] to project the KEGG pathways within the gut microbiome data across three cancer datasets. We integrated these predictions with the microbial clustering data and employed one-way ANOVA to identify specific pathways with significant differential enrichment across the subgroups. Subsequently, we determined the functional modules shared among CRC, GC, and BC. Then, the KEGG pathways that were also present in humans were retained. Finally, we screened the genes involved in these pathways and integrated cancer patient survival data from TCGA to identify gene sets significantly associated with survival.

#### Identification of TCGA-BRCA cancer sample subtypes and construction of the score model

Subtyping method: Cluster analysis was performed using the R ConsensusClusterPlus [64] package with a distance-based k-means algorithm, with the number of subsets (reps) set to 1000.

Scoring method [31]: For each sample, the score was calculated as ∑ (beta × Exp), where beta is the independent prognostic coefficient obtained through single-factor Cox regression analysis of the gene and Exp is the expression level of the gene.

Pathway enrichment analysis [65]: Gene set variation analysis (GSVA) is an algorithm building on gene set enrichment analysis (GSEA) that is available at http://www.gsea-msigdb.org/. Analysis of hallmark gene sets and pathways was conducted using the GSVA package in R. The limma package in R was used to identify significantly differentially expressed genes (DEGs) in pairwise comparisons. The R packages GSEABase, clusterProfiler, and org.Hs.eg.db were used for Gene Ontology (GO) and Kyoto Encyclopedia of Genes and Genomes (KEGG) pathway enrichment analyses of the differentially expressed genes. The Benjamini–Hochberg procedure was used to control the false discovery rate (FDR; p.adj) for multiple comparisons, and FDR < 0.05 was applied as the threshold for selection.

#### Single-cell transcriptome analysis

Sequencing data quality control and gene quantification: Raw data generated via high-throughput sequencing, in fastq format, were processed using the official 10x Genomics software Cell Ranger (version 7.0.1). This software allows the acquisition of data quality statistics and alignment to the reference genome (human: GRCh38, mouse: mm10). By identifying cell-specific barcode markers and unique molecular identifiers (UMIs) for each mRNA molecule within a cell, Cell Ranger quantifies high-throughput single-cell transcriptome data, calculating quality control statistics such as the number of high-quality cells, the median number of genes, and sequencing saturation.

Gene quantification quality control and data preprocessing: After preliminary quality control processing with Cell Ranger, additional quality control processing was performed using Seurat (version 4.0.0). Based on the distribution of indicators such as nUMI, nGene, and percent.mito, filtering criteria were applied to retain high-quality cells. The specific quality control criteria included retention of cells with a gene count of greater than 200, a UMI count of greater than 1000, a log10GenesPerUMI value of greater than 0.7, and a mitochondrial UMI count of less than 5%; and a percentage of red blood cells expressing a gene of less than 5%. Additionally, DoubletFinder software (version 2.0.3) was utilized to remove doublet cells. After quality control, the NormalizeData function in Seurat was applied for data normalization.

Dimensionality reduction and clustering analysis: The FindVariableGenes function (mean.function = FastExpMean, dispersion.function = FastLogVMR) in Seurat was used to select the top 2000 highly variable genes (HVGs). Principal component analysis (PCA) was performed using the expression profiles of the highly variable genes, and the results were visualized in two-dimensional space using uniform manifold approximation and projection (UMAP; a nonlinear dimensionality reduction technique).

Identification of marker genes: The FindAllMarkers function in Seurat (test.use = presto) was used for marker gene identification. This process allowed the identification of genes that were upregulated in each cell type compared to the other cell types, thus serving as potential marker genes. Visualization of the identified marker genes was performed with the VlnPlot and FeaturePlot functions.

Cell type identification: Via the SingleR package (version 1.4.1), the expression profiles of the cells to be identified were correlated with a common reference dataset. The cell type with the highest correlation in the reference dataset was assigned to the cells being identified, reducing subjective interference. The identification principle involved calculating the Spearman correlation coefficient between the expression profile of each cell in the sample and each annotated cell expression profile in the reference dataset, with the cell type with the highest correlation selected as the final identified type.

Differential gene expression and enrichment analyses: The FindMarkers function in Seurat (test.use = presto) was used to select differentially expressed genes. Genes with a P value less than 0.05 and a fold change greater than 1.5 were considered significantly differentially expressed. GO term and KEGG pathway enrichment analyses of the significantly differentially expressed genes were conducted using the hypergeometric distribution test.

#### Multiplex immunofluorescence staining

We conducted multiplex immunofluorescence (mIF) staining using antibodies specific for CD4 (rabbit monoclonal, clone EPR19514, Abcam, Cat# ab183685), CD8 (rabbit monoclonal, clone EPR21769, Abcam, Cat# ab217344), CCL5 (RANTES) (rabbit polyclonal, clone 25HCLC, Thermo Fisher, Cat# 710,001), CD103 (integrin alpha E)) (mouse monoclonal, clone 2E7, Thermo Fisher, Cat# 14-1031-82), and FoxP3 (rabbit monoclonal, clone D6O8R, Cell Signaling Technology, Cat# 12,653). Tissue sections were deparaffinized with xylene, rehydrated with ethanol, and subjected to antigen retrieval by boiling in Tris-EDTA buffer (pH 9.0) for 15 min. Endogenous peroxidase activity was blocked by incubation with 3% hydrogen peroxide at room temperature for 15 min. Nonspecific antigens were blocked by incubation with a goat serum solution for 30 min. The sections were then incubated with primary antibodies overnight at 4 °C and with horseradish peroxidase (HRP)-conjugated secondary antibodies at room temperature for 30 min. Subsequently, the sections were incubated with Opal tyramide signal amplification (TSA) fluorochromes (Opal Colour Manual IHC Kit, Perkin Elmer, NEL811001KT) at 37 °C for 20 min. Between each run, the antibody (Ab)-TSA complexes in the sections were removed by microwave heating, and the sections were blocked with a goat serum solution. In the final run, 4’,6-diamidino-2-phenylindole, dihydrochloride (DAPI) was added for visualization of nuclei, and the sections were mounted with glycerin.

#### Single-cell spatial transcriptome analysis

Sequencing data quality control and gene quantification: Raw data generated via high-throughput sequencing, in fastq format, were processed using the official 10x Genomics software Space Ranger (version 2.0.1) for the Visium spatial transcriptome sequencing data and bright-field microscopy slice images. The software detected the capture regions of tissues on the chip, aligned them to the reference genome (human: GRCh38, mouse: mm10), and, based on spatial barcode information, differentiated the reads for each spot. Statistical evaluations included the total spot count, reads per spot, detected gene count, and UMI count, providing an assessment of sample quality.

Gene quantification quality control and data preprocessing: After preliminary quality control processing with Space Ranger, further quality control and processing were performed using Seurat (version 4.3.0) [66]. The sctransform function was used to normalize the data, detect high-variance features, and store the data in the SCT matrix.

Dimensionality reduction and clustering analysis: The FindVariableGenes function n Seurat was used to select the top 3000 highly variable genes. PCA was conducted using the expression profiles of the highly variable genes, and the results were visualized in two-dimensional space using UMAP (nonlinear dimensionality reduction).

Identification of spatial feature genes: The FindAllMarkers function in Seurat (test.use = bimod) was employed for the identification of marker genes, revealing genes upregulated in each spot group compared to the other spot groups. These genes represented potential marker genes for each spot group, and visualization of the identified marker genes was performed with the VlnPlot and FeaturePlot functions.

Spatial cell type annotation: Robust cell type decomposition (RCTD) [67] (version 1.1.0) is a robust cell type deconvolution method that leverages cell type profiles obtained via single-cell RNA-seq to decompose mixtures of cell types while correcting for differences across sequencing techniques. For RCTD, the creat.RCTD function was used with default parameters, ensuring at least 1 cell per cell type and at least 1 UMI per spot. The run.RCTD function was used with doublet_mode set to FALSE, allowing the cell type composition of each spot to be inferred.

Differential gene expression and enrichment analyses: The FindMarkers function of Seurat was used for selection of differentially expressed genes, and genes with a P value less than 0.05 and a fold change greater than 1.5 were identified by filtering. GO term and KEGG pathway enrichment analyses of the significantly differentially expressed genes were conducted using the hypergeometric distribution test.

#### PDX mouse models and drug treatment

Tumour tissues isolated from patient YZL_1 were dissected into 1-mm3 pieces. After NOG mice were anaesthetized, the BC tissues were subcutaneously implanted into the right superior flank. When the tumour diameter reached 1 cm (approximately 60 days after transplantation), we removed the subcutaneous PDX tumours, dissected them into 3 pieces of approximately 2 × 2 × 2 mm each, and then retransplanted the pieces into the flanks of the nude mice to allow tumour growth for approximately one month. The mice were euthanized after no more than 5 weeks or when the tumour diameter reached 10 mm. Beginning on the seventh day after transplantation, each mouse in the drug treatment groups received 20 mg/kg sonidegib or 10 mg/kg rapamycin every two days via tail vein injection. Beginning on the seventh day after transplantation, each mouse in the control group received placebo every two days via tail vein injection.

#### Subtype identification based on TCGA classification

Following the classification process, we employed the random forest algorithm using the R software package library(randomForest) to develop a predictive model based on the gene expression profiles and classification data. This model had predictive capability, allowing the input of expression profile data from new datasets to determine the corresponding classification outcomes.

For the single-cell sequencing data, we first screened the expression of the 700 genes associated with gut microbiota-related metabolic pathways and patient survival within each cell and determined their average expression levels.

#### Statistical analysis

Student’s t test and the Mann‒Whitney test were applied to compare continuous variables and categorical variables, respectively, where appropriate. The associations between clinical information and metabolic pathway-based subtypes were examined using the chi-square test and Fisher’s exact test. Survival curves were constructed using the Kaplan‒Meier method and compared with the log-rank test. Univariate and multivariate Cox proportional hazards regression models with or without adjustment for available prognostic clinical covariates were used to calculate hazard ratios (HRs) and 95% confidence intervals. Correlations were analysed with Spearman correlation analysis. All the statistical analyses were performed with R software or GraphPad Prism software.

### Electronic supplementary material

Below is the link to the electronic supplementary material.

Supplementary Material 1

Supplementary Material 2

## Data Availability

No datasets were generated or analysed during the current study.
