# Supplementary material for: Multiomics-based molecular subtyping based on the commensal microbiome predicts molecular characteristics and the therapeutic response in breast cancer
Source: Mol Cancer. 2024 May 10;23:99. doi: 10.1186/s12943-024-02017-8 (PMC11083817; doi:10.1186/s12943-024-02017-8)
Supplement: Supplementary file 1 — Supplementary Material 1 [file 12943_2024_2017_MOESM1_ESM.docx]

**Supplementary figures**

**
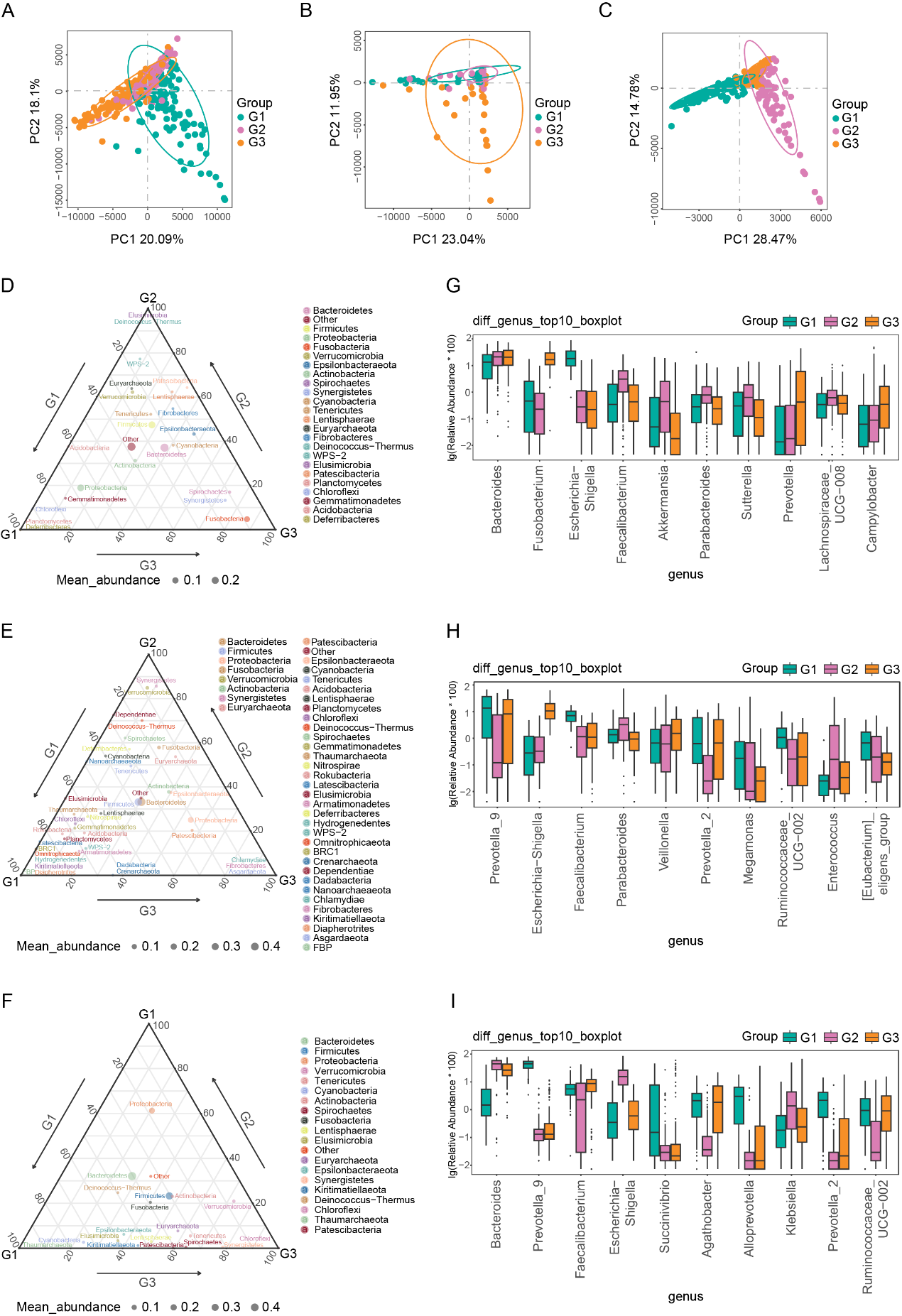
**

**Figure S1. Microbial feature analysis of clusters in breast cancer (BC), gastric cancer (GC) and colorectal cancer (CRC).** (A-C) Principal coordinate analysis was performed based on the Bray-Curtis distance matrix of the clusters in CRC, GC and BC. (D-F) Abundance schematic of each phylum in the three clusters in CRC, GC and BC. Closer proximity to a specific cluster indicates higher abundance in that cluster. (G-I) The boxplot of top 10 significantly different genera in CRC, GC and BC.


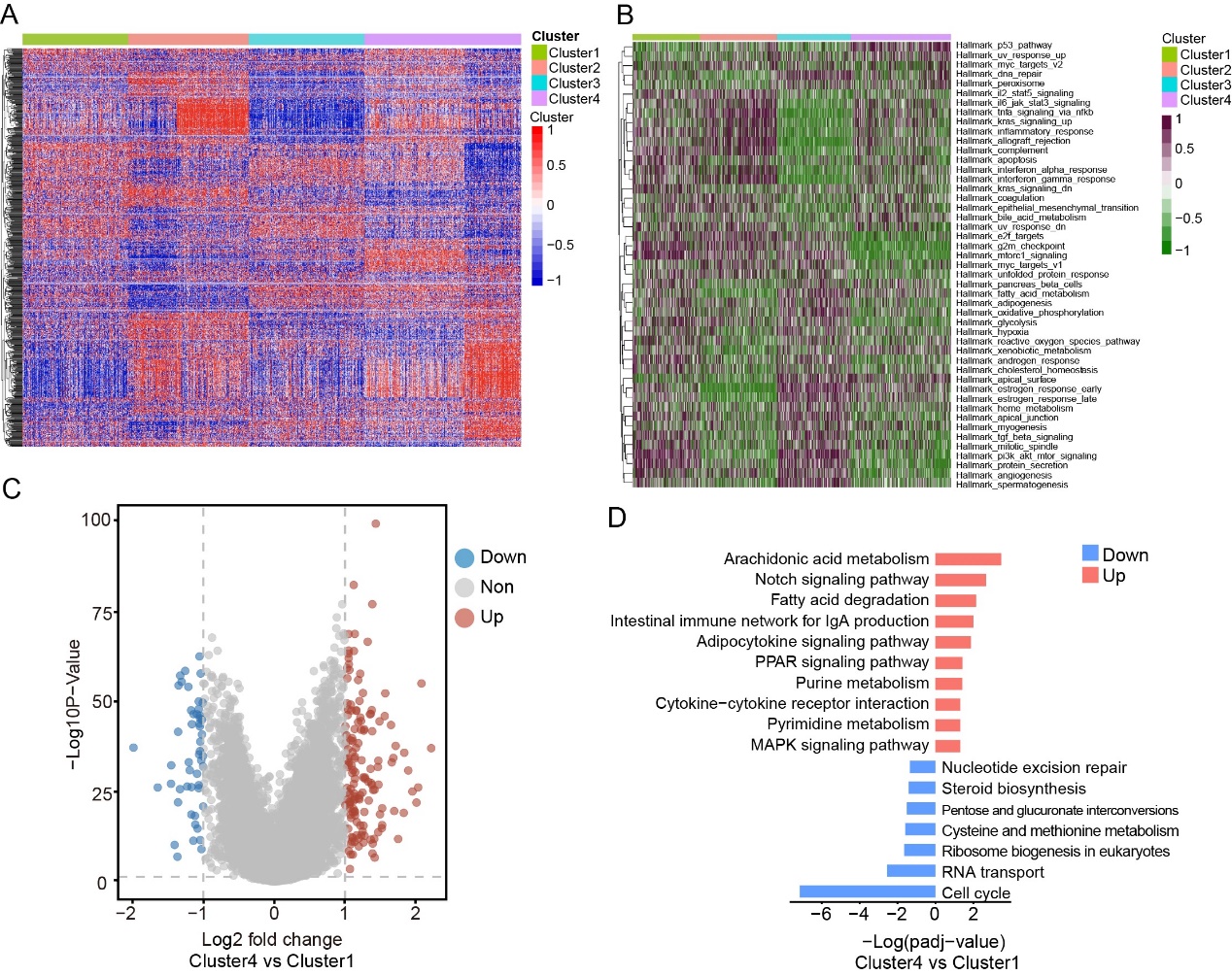


**Figure S2. The analysis of differential metabolic pathways in BC clusters.** (A) A distance-based k-means method was employed to categorize samples into four clusters. (B) Heatmap of differential metabolic pathways in clusters. (C) The volcano plot of differentially expressed genes between cluster 1 and cluster 4. (D) The bar plot of differential pathways between cluster 1 and cluster 4. A significant enrichment of immune-related pathways in Cluster 4. *** P < 0.001. **P < 0.01. *P < 0.05.


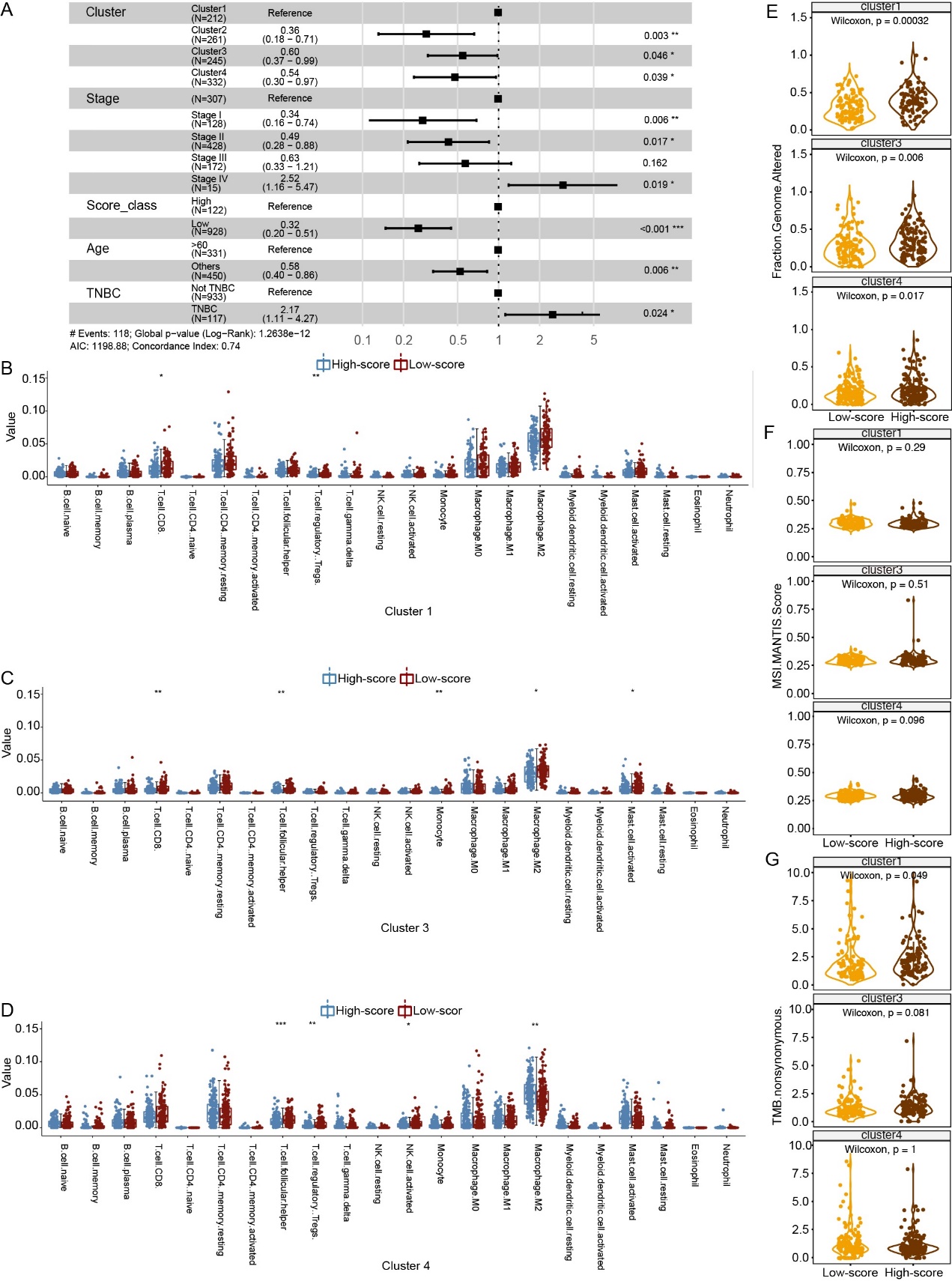


**Figure S3. The comparison of immune cell expression in clusters between the high-score and low-score groups**. (A) Multivariate Cox proportional regression analysis of factors associated with OS. (B) The comparison of immune cell expression in Clusters 1 between the high-score and low-score groups. (C) The comparison of immune cell expression in Clusters 3 between the high-score and low-score groups. (D) The comparison of immune cell expression in Clusters 4 between the high-score and low-score groups. (E-G) The boxplot of fraction genome altered, TMB, MSI MANTIS score and TMB nonsynonymous between high-score and low-score groups for BC clusters at the genetic level. *** P < 0.001. **P < 0.01. *P < 0.05.


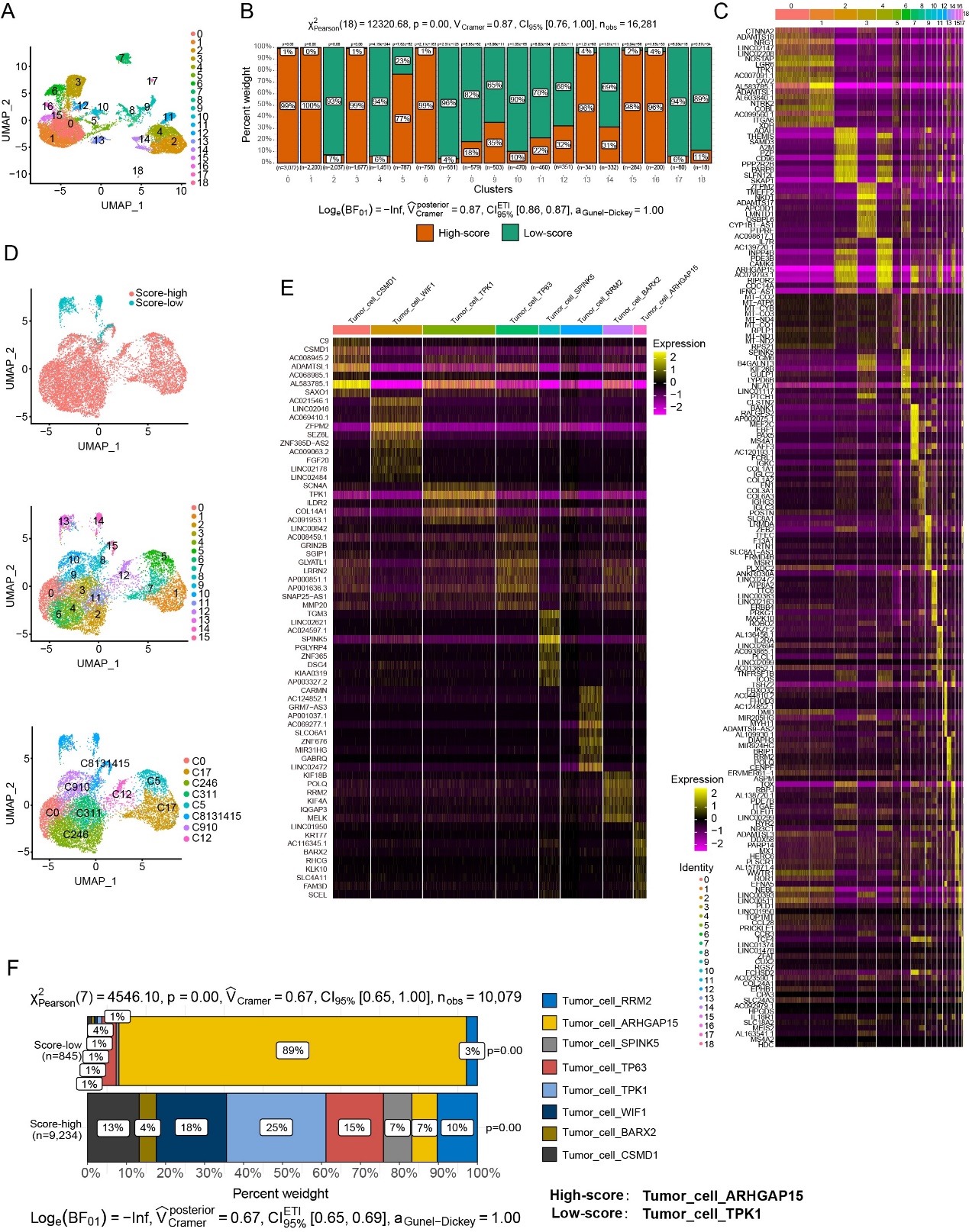


**Figure S4. Single-cell level cell cycle and tumor cell annotation analysis of challenging BC.** (A) UMAP plot showing 19 cell types identified by integrated analysis in all the clusters of single-cell sequencing data. (B) Proportions and chi-square tests for high-score and low-score in each cell types. (C) Heatmap showing the expression of marker genes in each cell types. The top bars label the clusters corresponding to specific cell types. (D) UMAP plot showing different score in all tumour cells. 16 types of tumour cells were annotated and ultimately classified into eight tumour subgroups. (E) Heatmap showing the expression of marker genes in the indicated tumour cell types. The top bars label the clusters corresponding to specific tumour cell types. (F) Proportions and chi-square tests for high-score and low-score in each tumour cell types. In the low-score group, 89% of the cells were identified as tumour cell ARHGAP15. In the high-score group, 25% of the cells were tumour cell TPK1.


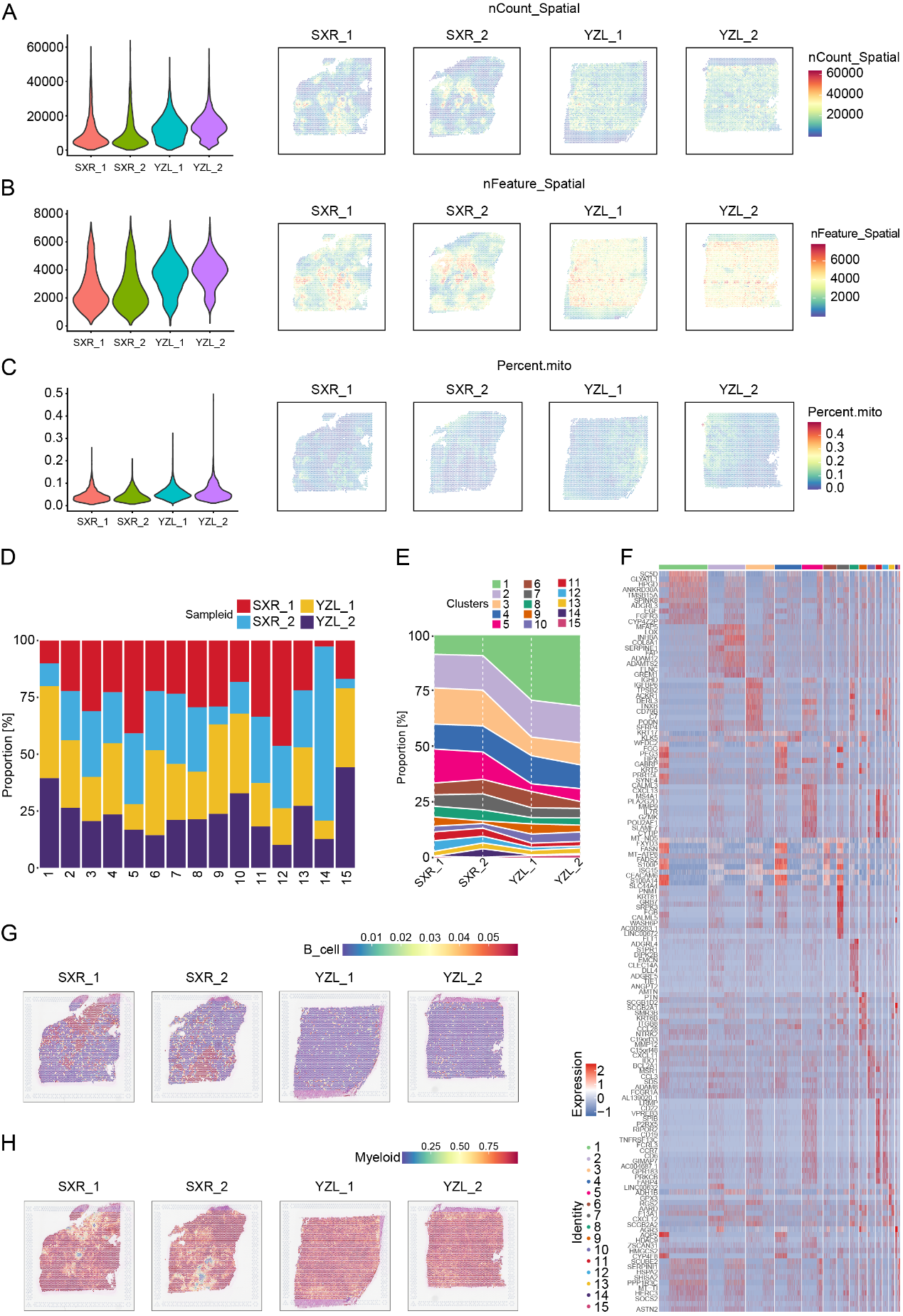


**Figure S5. Single-cell spatial transcriptomic analysis of challenging BC.** (A-C) The results of quality control of Single-cell spatial transcriptomic sequencing to acquire in situ gene expression profiles of four patients with TNBC. (D-E) The proportion of 15 different cell types annotated in four patients. (F) Heatmap showing the expression of marker genes in the indicated cell types. The top bars label the clusters corresponding to specific cell types. (G-H) The expression of B cells and myeloid cells were analyzed, along with their marker genes, finding a similar pattern with more abundance in the low-score group, consistent with earlier results.


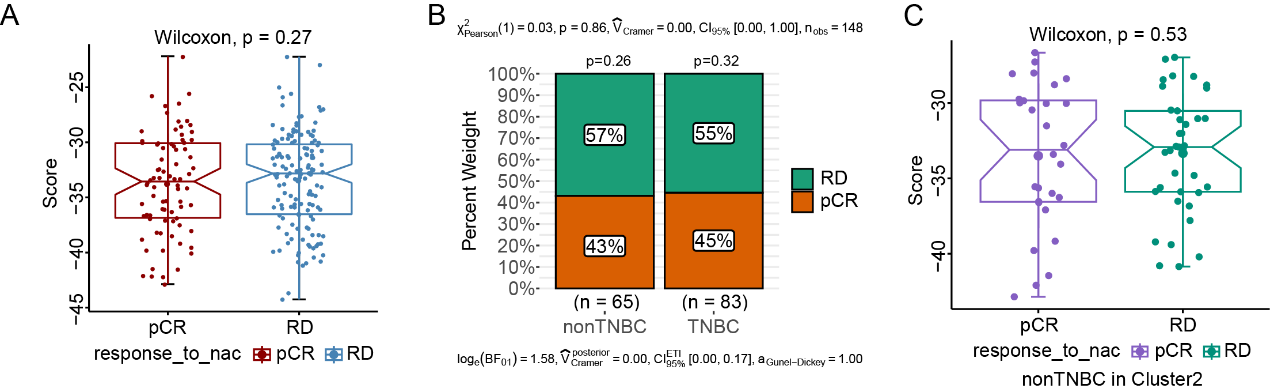


**Figure S6. The characters of pathologic complete response (pCR) and the residual disease (RD) in all samples and nonTNBC.** (A) Scores were calculated for each sample and observed no significant differences in score distribution between pCR and RD groups. (B) Proportions and chi-square tests for RD and pCR in nonTNBC and TNBC groups. The distribution of RD and pCR not show significant differences. (C) The boxplot of score between pCR and RD groups in nonTNBC samples. There was no significant difference between pCR and RD.

**Supplementary tables**

Table1: Relative abundance of microbial genera in CRC samples

Table2: Relative abundance of microbial genera in GC samples

Table3: Relative abundance of microbial genera in BC

Table4: Clustering information of microbial tumour samples

Table5: Selected KEGG pathways

Table6: Uni_cox results for 700 genes

Table7: Subtype information and scoring information for TCGA genes

Table8: Cell type identification table for single-cell two samples

Table9: Treatment cohort classification information
